# Gray Matter Atrophy in Parkinson’s Disease and the Parkinsonian Variant of Multiple System Atrophy: A Combined ROI- and Voxel-Based Morphometric Study

**DOI:** 10.6061/clinics/2020/e1505

**Published:** 2020-06-08

**Authors:** Xiaorui Cui, Lan Li, Lei Yu, Huijuan Xing, Hong Chang, Li Zhao, Jin Qian, Qingwei Song, Shiyu Zhou, Chunbo Dong

**Affiliations:** I Department of Neurology, Affiliated Hospital of Xiangnan University, Chenzhou, China; II Department of Neurology, Second Affiliated Hospital of Dalian Medical University, Dalian, China; III Department of Neurology, Dalian Friendship Hospital, Dalian, China; IV Department of Neurology, The Third People’s Hospital of Dalian, Dalian, China; V Department of Neurology, First Affiliated Hospital of Dalian Medical University, Dalian, China; VI Department of Radiology, First Affiliated Hospital of Dalian Medical University, Dalian, China; VII Department of Psychology, Dalian Medical University, Dalian, China

**Keywords:** Parkinson’s Disease, Multiple System Atrophy, Magnetic Resonance Imaging, Regions of Interest, Voxel-Based Morphometry, Gray Matter Atrophy

## Abstract

**OBJECTIVES:**

Parkinson’s disease (PD) and the parkinsonian variant of multiple system atrophy (MSA-P) are distinct neurodegenerative disorders that share similar clinical features of parkinsonism. The morphological alterations of these diseases have yet to be understood. The purpose of this study was to evaluate gray matter atrophy in PD and MSA-P using regions of interest (ROI)-based measurements and voxel-based morphometry (VBM).

**METHODS:**

We studied 41 patients with PD, 20 patients with MSA-P, and 39 controls matched for age, sex, and handedness using an improved T1-weighted sequence that eased gray matter segmentation. The gray matter volumes were measured using ROI and VBM.

**RESULTS:**

ROI volumetric measurements showed significantly reduced bilateral putamen volumes in MSA-P patients compared with those in PD patients and controls (*p<*0.05), and the volumes of the bilateral caudate nucleus were significantly reduced in both MSA-P and PD patients compared with those in the controls (*p<*0.05). VBM analysis revealed multifocal cortical and subcortical atrophy in both MSA-P and PD patients, and the volumes of the cerebellum and temporal lobes were remarkably reduced in MSA-P patients compared with the volumes in PD patients (*p<*0.05).

**CONCLUSIONS:**

Both PD and MSA-P are associated with gray matter atrophy, which mainly involves the bilateral putamen, caudate nucleus, cerebellum, and temporal lobes. ROI and VBM can be used to identify these morphological alterations, and VBM is more sensitive and repeatable and less time-consuming, which may have potential diagnostic value.

## INTRODUCTION

Multiple system atrophy (MSA) is a sporadic, adult-onset disease predominantly characterized by motor symptoms, such as varying degrees of parkinsonism and cerebellar ataxia. MSA has two subtypes: the parkinsonian variant (MSA-P) and the cerebellar variant (MSA-C). Clinical differentiation between Parkinson’s disease (PD) and MSA-P remains a challenge for neurologists. According to the existing literature, some scholars have proposed that magnetic resonance T2*-weighted gradient echo imaging and diffusion-weighted imaging are of diagnostic value for differentiating MSA-P from PD (1,2). To date, the morphological differences between MSA-P and PD have yet to be fully understood. Recently, magnetic resonance imaging (MRI) has been extensively used in brain morphometry. Volumetric analytical methods include manual measurement of the region of interest (ROI) and voxel-based comparisons of anatomic data, especially voxel-based morphometry (VBM) (3). However, brain morphological studies designed to compare MSA with PD have not yielded consistent results (4). Ghaemi et al. proposed that MSA-P differed significantly from PD in terms of decreased putaminal volume and postsynaptic D2 receptor density, and neither MRI volumetry nor PET imaging of the midbrain region contributed to differential diagnosis between PD and MSA-P (5). Feng et al. found that signal alterations in the putamen on T2-weighted MRI, including slit-like hyperintense rim and putaminal hypointensity, are not specific signs for differentiating MSA-P from PD. The specificity and sensitivity of putaminal atrophy for distinguishing MSA-P from PD were 92.3% and 44.4%, respectively (6). Barbagallo et al. compared the nigro-striatal changes between PD and MSA-P using multimodal MRI, and they noted that MSA-P was associated with higher mean diffusivity values in the putamina compared with PD. The putamina in patients with MSA-P had higher T2* relaxation rates than those in patients with PD. Combined evaluation of T2* relaxation rates and mean diffusivity in the putamen allowed 96% accuracy in differentiating PD from MSA-P (7). The aim of this study was to evaluate gray matter atrophy in PD and MSA-P using ROI-based morphometric measurement and VBM.

## METHODS

### Subjects

A total of 41 patients with PD (17 men and 24 women) and 29 patients with MSA-P (13 men and 16 women) were enrolled between September 2009 and March 2013. Thirty-nine age- and sex-matched healthy volunteers (20 men and 19 women) were recruited as normal controls; statistical analysis showed no significant difference in age or sex between patients and controls. Whole-brain anatomical MRI was performed with a 1.5-Tesla scanner (Siemens Medical System, Erlangen, Germany). Nine patients with MSA-P (three men and six women) were excluded due to poor imaging quality. This study was approved by the Ethics Committee of the First Affiliated Hospital of Dalian Medical University. All procedures performed in the study involving human participants were in accordance with the ethics standards of the institutional and national research committee and with the 1964 Helsinki Declaration and its later amendments or comparable ethics standards. Written informed consent was obtained from all individual participants included in the study.

### MRI acquisition

MRI data were acquired on a 1.5T GE HD Signa MR scanner. A three-dimensional fast spoiled gradient-echo sequence (3D-FSPGR) was used for the acquisition of 160-190 continuous T1-weighted slices of 1.0 mm thickness in the sagittal plane. Imaging parameters were as follows: repetition time (TR)=9.6 ms; echo time (TE)=4.2 ms; flip angle=15°; number of excitations (NEX)=1; field of view (FOV)=256 mm; slice thickness=1 mm; slice interval=0 mm; matrix size=256×256; and voxel size=1.0×1.0×1.0 mm.

### ROI-based morphometric measurements

The image processing system for qualitative and quantitative volumetric ROI analysis has been previously described (8). Briefly, all raw data were transferred to a Unix workstation (Silicon Graphics, Mountain View, CA, USA), and the image data was processed using the software package Dr View 5.0 (Asahi Kasei Joho System, Tokyo, Japan). Prior to the formal measurement, 15 subjects were randomly selected for the evaluation of intergroup consistency, and two evaluators performed ROI analysis independently in a double-blind manner. Brain images were realigned in three dimensions and reconstructed into continuous coronal slices of 1 mm thickness perpendicular to the anterior commissure-posterior commissure line. The whole cerebrum was separated from the brainstem and cerebellum. The signal intensity histogram distributions across the whole cerebrum were used to segment the voxels semiautomatically into gray matter, white matter, and cerebrospinal fluid (CSF). The intracranial volume (ICV) was also measured. Manual volumetric measurements were performed on the ROIs (putamen, caudate nucleus, and pallidal globus) in bilateral hemispheres using consecutive 1 mm coronal slices, with the corresponding sagittal and axial planes presented simultaneously for the assurance of landmarks and the integrity of the ROIs.

#### Putamen:

The entire putamen was manually traced and was bounded laterally and anteriorly by the external capsule and separated from the rest of the striatum by a line extending inferiorly from the anterior limb of the internal capsule and from the globus pallidus to the lateral medullary lamina.

#### Caudate:

The head and body of the caudate were manually traced. To separate the caudate from the nucleus accumbens, a line was drawn from the most inferior point of the lateral ventricle to the most inferior medial point of the internal capsule.

#### Globus pallidus:

The entire globus pallidus was bounded superiorly and medially by the internal capsule and inferiorly by the substantia innominata and anterior commissure.

Two trained raters (Cui and Zhou), who were blinded to the subjects’ identities, measured the volumes of the ROIs respectively. Inter- and intrarater intraclass correlation coefficients for 15 randomly selected brains were over 0.95 for the ROIs.

Statistical analyses were performed using multivariate analysis of covariance (MANCOVA) with repeated measures via STATISTICA 6.0 software, and ICV and age were set as covariates for each region (9). The intergroup variants included diagnosis (MSA-P, PD, or normal) and gender (male or female), and the hemispheric side (right or left) was used as the intragroup variant for comparing the specific ROI volumes. Post hoc Tukey’s HSD tests were used for analyzing the significant main effects or significant interaction effects yielded by MANCOVA by which pairwise group differences (ROI-to-ROI) were checked. Analysis of covariance (ANCOVA) was used to compare the gray matter volumes with the ICV and age as covariates. The age and education level in the three groups were compared using one-way analysis of variance. The distribution of gender was compared using the chi-square test. The UPDRS part III scores and improved H-Y grading scores were compared using the t test. Pearson’s partial correlation was used to analyze the correlation between brain volume and clinical symptoms. Statistical significance was defined as *p*<0.05 (two-tailed).

### Image processing using VBM

VBM was performed in the SPM8 software package (http://www.fil.ion.ucl.ac.uk/spm/). All T1-weighted images were spatially normalized in the standardized space using the template provided by the Montreal Neurological Institute (10). Then, all images were segmented into gray and white matter as well as CSF. The images were subsequently modulated with Jacobian determinants to compensate for volume changes in nonlinear spatial normalization (11). Finally, the images were smoothed with a Gaussian filter of 8×8×8 mm full width at half maximum (FWHM). Using SPM8, ANOVA was performed to compare the data in the three groups, and the results were saved as a mask; then, independent-sample t tests were performed for post hoc analyses in which the previously generated mask was imported. During this procedure, comparisons between groups were performed between brain regions that showed significant differences in the ANOVA. The age and sex of each subject were entered into the design matrix as nuisance variables. The global volumes in the voxel intensities were used as confounding covariates, and anatomy-based templates were used for subcortical regions of specific interest (putamen, caudate and globus pallidus), which originated in the Automated Anatomical Labeling (AAL) map of MRicro (http://www.cabiatl.com/mricro/mricro/index.html) (12).

Expected voxels per cluster generated in the SPM processing were documented. Statistical significance for cluster selection in the striatum between MSA-P patients and controls was set at *p*<0.001 (uncorrected), with an extended threshold of 114 voxels. The comparison of whole cerebral gray matter between MSA-P patients and controls was performed at a threshold of *p*<0.05 (voxel level FWE), with an extended threshold of 16 voxels. Significance was set at *p*<0.001 (uncorrected) for comparisons of MSA-P *vs* PD groups and PD *vs* control groups in both the striatum (extended threshold of voxel >0) and whole cerebral gray matter (extended threshold of voxel >75). The anatomical position of each cluster via T (or F) test images was performed using Xjview software (http://www.alivelearn.net/xjview8/).

### Clinical assessment and correlation analysis

The clinical severity for MSA-P and PD patients was independently assessed according to parts III and V (modified Hoehn-Yahr staging scale) of the Unified Parkinson’s Disease Rating Scale (UPDRS) by two neurologists (13). The association between clinical severity and gray matter atrophy was evaluated using partial correlations analysis.

## RESULTS

### Demographic and clinical features

The demographic data of the subjects are summarized in Table 1. There were no significant differences in age (*p*=0.22), sex (*p*=0.65), or educational time (*p*=0.73) between the patients and controls. There was no significant difference in the duration of disease between MSA-P and PD patients (*p*=0.32). The patients with MSA-P had significantly higher UPDRS part III scores (*p*=0.04) and part V scores (*p*<0.01) than those of patients with PD.

### ROI results

**Figure 1:**
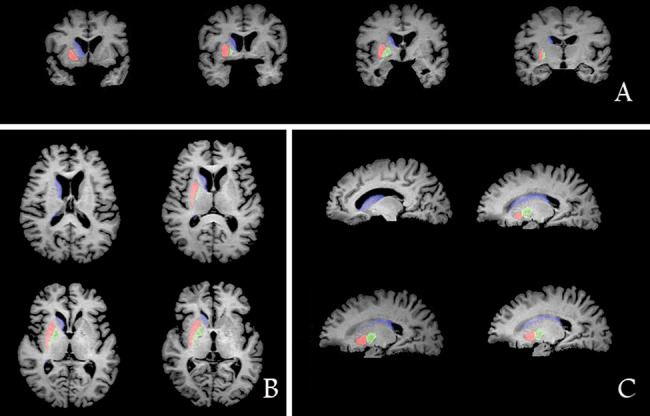
Manual ROI measurements (A, coronal; B, axial; C, sagittal) showing the putamen (red), caudate nucleus (blue) and globus pallidus (green).

The correlation coefficients for intergroup consistency in the measurement of ICV, putamen volume, caudatum volume, and pallidum volume were 0.99, 0.98, 0.99, and 0.95, respectively. Comparisons of the striatum volume and whole cerebral gray matter volume among the MSA-P patients, PD patients, and healthy controls are shown in Figure 1.

The whole cerebral gray matter volume was significantly smaller in the MSA-P patients compared with that of the controls (main effect of group in ANCOVA; *F*=4.91, *p*<0.01; post hoc test, *p*<0.01). The PD patients had significantly smaller gray matter volumes compared with those of the controls (*p*<0.01).

The MANCOVA for ROIs revealed the bilateral putamen (*F*=3.50, *p*<0.01) and bilateral caudate nucleus (*F*=5.50, *p*<0.001) were the main effects associated with distinct diagnoses. Post hoc tests showed that the bilateral putamen volume was smaller in MSA-P patients than that in normal controls (*p*=0.006) and that in PD patients (*p*=0.01). Post hoc tests indicated that the bilateral caudate nucleus volume was smaller in MSA-P patients compared with that in normal controls (*p*=0.002). The patients with PD had significantly decreased volumes in the bilateral caudate nucleus (*p*<0.001) compared with those of the controls.

### VBM results

#### Striatal regions:

**Figure 2:**
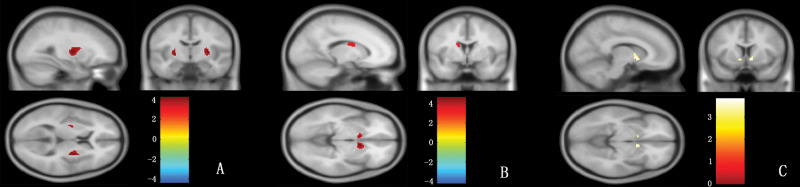
VBM analysis of the putamen, caudate nucleus and globus pallidus. In this figure, the red color represents the volumes of the bilateral putamen in controls that are larger than MSA-P (A) and the volumes of the bilateral caudate nucleus in controls that are larger than MSA-P (B); the yellow color represents the volumes of the bilateral caudate nucleus in controls that are larger than PD (C), *p* (uncorrected) <0.001.

Compared with normal controls, the gray matter volumes of the bilateral caudate nucleus and bilateral putamen were significantly reduced in patients with MSA-P (Figure 2A,B); however, only the volume of the bilateral caudate nucleus was reduced in patients with PD (Figure 2C). For the gray matter volume of striatal regions, there was no significant difference between MSA-P and PD patients.

#### Cortical and other subcortical regions:

**Figure 3:**
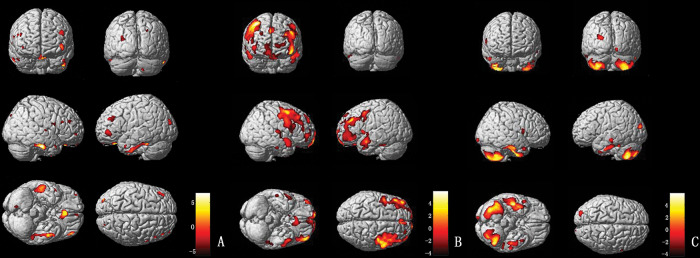
(A) VBM analysis of the gray matter comparing MSA-P and controls. The yellow-white color represents the gray matter volume (controls>MSA-P); the brown-dark color indicates the gray matter volume (MSA-P>controls). (B) VBM analysis of the gray matter comparing PD and controls. The yellow-white color represents the gray matter volume (controls>PD); the brown-dark color indicates the gray matter volume (PD>controls). (C) VBM analysis of the gray matter comparing MSA-P and PD. The yellow-white color represents the gray matter volume (PD>MSA-P); alternatively, the brown-dark color marks the gray matter volume (MSA-P>PD).

MSA-P patients (*vs* controls) exhibited severe gray matter atrophy in the temporal and frontal lobes, including the bilateral inferior temporal gyrus, the bilateral superior temporal gyrus, and the bilateral gyrus rectus. In addition, parts of the bilateral thalamus, occipital lobes, and cerebellum were also involved (Figure 3A). PD patients (*vs* controls) exhibited gray matter atrophy in the bilateral middle frontal gyrus, right orbit frontal gyrus and left middle temporal gyrus. Moreover, parts of the left thalamus and frontotemporal lobes were also involved (Figure 3B). Compared with PD patients, the most remarkable gray matter atrophy in the MSA-P patients was identified in the cerebellum (the bilateral cerebellum posterior lobe and the left cerebellar tonsil) and the temporal lobes (the bilateral inferior temporal gyrus and the bilateral middle temporal gyrus). Several additional mild atrophic regions were identified in the occipital and frontal lobes (Figure 3C).

### Clinical-radiological correlation

The results of correlation analysis between UPDRS part III and V scores are summarized in Table 2.

In patients with MSA-P, the UPDRS part III scores were negatively correlated with gray matter atrophy in the left putamen (*r*=-0.592, *p*=0.01), and there was no correlation between the UPDRS part III scores and atrophy in the right putamen, bilateral caudate nucleus, globus pallidus, or whole-brain gray matter (all *p*>0.05). The UPDRS part V (modified Hoehn-Yahr staging scale) scores were negatively correlated with gray matter atrophy in the left putamen (*r*=-0.532, *p*=0.023), and there was no correlation between the UPDRS part V scores and atrophy in the right putamen, bilateral caudate nucleus, globus pallidus, or whole-brain gray matter (all *p*>0.05). The disease durations were negatively correlated with gray matter atrophy in the left putamen (*r*=-0.577, *p*=0.012) and in the right putamen (*r*=-0.722, *p*=0.001). Additionally, the disease durations were positively correlated with the UPDRS part III scores (*r*=-0.597, *p*=0.009) but not with the UPDRS part V scores (*p*>0.05).

In patients with PD, the correlation of gray matter volume with the UPDRS part III scores and modified H&Y scores are shown in Table 2. There was no correlation between the UPDRS part III or modified H&Y scores and the gray matter volume (whole-brain gray matter volume, putamen volume, caudatum volume or pallidum volume) (all *p*>0.05). The disease duration showed a tendency of negative correlation with gray matter volume, although there was no significant difference (*p*>0.05). The disease duration had no correlation with the UPDRS scores (*p*>0.05).

## DISCUSSION

### Structural alterations and pathophysiology of disease

To our knowledge, this is the first study to compare striatal and whole brain gray matter volumes in MSA-P and PD patients using a manual ROI-based volumetric analysis and an automated VBM method. Previously, only one type of volume measurement method has been used to evaluate changes in brain structure, and the results varied from previous pathological findings (14). In this study, both the ROI and VBM results indicated a reduction in the bilateral putamen volume in the MSA-P group compared with those of the PD and healthy control groups. This finding is consistent with previous studies (15). The decreased volume was significantly correlated with movement disability in the disease process (16), which was apparent from the PD and healthy controls. The evidence indicated that the degeneration of selective interneurons occurred in early MSA and was associated with pathological damage and the formation of inclusion bodies. However, little evidence suggests the presence of neuronal degeneration and loss in PD. The compensatory morphological changes in striatal interneurons occurred only during PD progression, and the dopamine replacement therapy effects were no longer ideal (17). The occurrence of parkinsonian syndrome in MSA is directly related to the degeneration and loss of neurons in the putamen.

The current study demonstrated that the caudate nucleus mainly receives afferent information from regions related to higher nervous and emotional activities, and the integration of the afferent inputs may be related to the initiation of voluntary movement. Limited evidence supports reduced metabolism and relaxation time in the caudate nucleus of MSA-P, which thereby promotes damage to the caudate nucleus (18). Our study identified significant atrophy in the caudate nucleus of the MSA-P group, which is consistent with previous pathologic and VBM results (19). This finding provides the morphological basis for the pathophysiology of caudate nucleus damage. The caudate nucleus in PD also decreased in this study, which is consistent with the MRI results obtained by Lisanby et al. (20). Some studies have confirmed that the caudate nucleus damage is related to the cognitive behavior; however, other detailed investigations regarding Parkinson’s disease with dementia (PDD) have resulted in different conclusions (21). Cognitive assessments were not performed in our research. Therefore, the relationship between cognitive function and pathological changes in the caudate nucleus has yet to be confirmed.

Fewer studies have measured the globus pallidus volume. Atrophy was not present in the bilateral globus pallidus of the three groups in our study, which is consistent with Hardman’s findings regarding PD and PSP (22). In general, the globus pallidus is intact in PD; however, this finding is in contrast to the results of O’Neill et al. (23), which indicated that the globus pallidus was atrophied by 16% in PD patients compared with normal age-matched controls (*p*<0.05). Variations in research methodology and race, as well as the disease periods of the included patients, may have influenced the results. In MSA patients, neurodegeneration is associated with disease development, which leads to the reduction of dopamine receptors in the basal ganglia and ultimately the development of levodopa-insensitive parkinsonian syndromes. According to the new pathological grading standards for MSA-P, which were formulated by Wenning et al. (24), the loss of globus pallidus neurons only appears at striatonigral degeneration (SND) level III, which is characterized by lateral atrophy.

Gilman et al. noted cortical and subcortical cholinergic deficits in patients with PD, MSA-P and PSP, suggesting functional and structural alterations of cortices and subcortices (25). In the current study, VBM analysis showed cortical and thalamic atrophy in patients with PD and MSA-P, which supports the above hypothesis.

The thalamus receives projections from the motor cortex. Specifically, the ventral thalamus transmits executive information to the anterior motor cortex with the participation of the basal nucleus. The ventrolateral area of the anterior thalamus transmits information to the primary motor area under the control of the basal nucleus and cerebellum, and the ventrolateral area of the posterior thalamus transmits feedback from the primary motor area under the regulation of the cerebellum. This cortical-striatal-thalamic-cortical connection constitutes an integrated circuit controlling movement. Our findings showed MSA-P is associated with bilateral thalamic atrophy, which is consistent with previous evidence (26). The longitudinal VBM study performed by Brenneis et al. revealed that thalamic atrophy is related to the course of MSA (27). These results support that the degeneration and loss of thalamic neurons may be involved in the pathophysiological process of MSA-P. Additionally, some scholars found that the norepinephrine level in the thalamus was decreased (28). The ventrolateral nucleus of the thalamus is closely related to the symptoms of static tremor of PD; the damage in the projection fibers from the substantia nigra to the thalamus can lead to a decrease in dopamine uptake in the ipsilateral striatum, resulting in static tremor symptoms in unilateral limbs. According to our VBM results, there was atrophy in the left thalamus, which may be associated with the pathophysiological process of PD. The definitive pathological changes in the thalamus and the correlation with motor symptoms in PD still need further research.

Additionally, we noted remarkable atrophy of the cerebellum in patients with MSA-P, which was consistent with previous reports (29). These findings indicate that degeneration of the olivary pontine cerebellopontine system is involved in the pathological process of MSA-P (30). Furthermore, we found that atrophy of the cerebellum and temporal lobe in the MSA-P group was more marked than that in the PD group, which may provide a new approach for differential diagnosis of MSA-P and PD. MSA is a neurodegenerative disease characterized by motor symptoms, memory deficits, emotional abnormalities and cognitive disorders. Our findings indicate that multiple cerebral cortices and circuits are involved in the pathophysiological process of MSA.

Existing evidence shows that the volume of the primary motor area and supplementary motor area was reduced in MSA-P (19), suggesting that the motor symptoms may be aggravated by the loss of neurons in these areas (4). Another VBM study showed atrophy of the insular lobe, orbital gyrus, and superior temporal gyrus in patients with MSA, and the memory impairment was significantly correlated with atrophy of the prefrontal lobe (12). These findings suggest that cortical atrophy is involved in the progression of MSA (27), which may lead to functional disorders and predict deterioration of the disease (12).

As is well known, the decrease of dopamine uptake in the substantia nigra striatum system is the major cause of PD. In patients with PD, the dysfunction in the sensorimotor loop may lead to compensative activation of the cortex-striatum-thalamus-cortex loop (31). Some scholars found that advanced function was damaged and phantoms appeared in patients with progressive PD, which may be related to the impaired function of the anterior cingulate gyrus (32). In recent years, there have been many studies on PD-related cognitive dysfunction (33), revealing that cognitive impairment and even dementia occur in 75% of all patients with PD. Cortical atrophy, decreased metabolism, white matter changes, dopamine/cholinergic uptake imbalance, and excessive deposition of amyloid substances may be possible mechanisms. Moreover, a recent positron emission tomography (PET) study in PD patients without cognitive impairment and dementia showed that the frontal lobe, temporal lobe, and cerebellum were associated with emotional and cognitive regulation, which may lead to apathy in PD. We also noted atrophy of the frontal lobe and temporal lobe in PD patients by VBM analysis, while the relationship between gray matter atrophy and cognitive/emotional impairment requires further evidence.

Pohjalainen et al. observed the density and affinity of dopamine (D_2_) receptors in normal striatum using PET, revealing that the affinity of D_2_ receptors in the left striatum in women was lower than that in men. These findings suggested that there is a high concentration of dopamine in the striatal system in women (34). A number of subsequent PET studies supported this view that the density of dopamine transporters and the ability to uptake dopamine in women were higher than those in men (35). This provides a pathophysiological basis for gender-related symptom differences and different responses to levodopa in patients with PD. In recent years, PET studies on PD have shown that the uptake of fluorodopa in the putamen and prefrontal lobe in women is higher than that in men, which indicates that there are also gender-related differences in dopamine function in PD. In a large cohort study of 230 patients with MSA, Watanabe et al. pointed out that gender was uncorrelated with clinical symptoms and progression of PD (36). However, O'Sullivan et al. found that the prognosis of PSD and MSA was worse in women (37). The inconsistent conclusions may be due to different races and variable evaluation methods. To date, the correlation between gender and striatal volume remains unclear. In the current study, we found there was no significant correlation between gender and striatum volume, suggesting the functional alterations may not be necessarily accompanied by structural changes.

Most of the patients with PD had unilateral onset, which has been confirmed by many PET and single photon emission computed tomography (SPECT) studies (38). The metabolism of the striatum was decreased in patients with PD and MSA, and the asymmetry of PD was more significant than that of MSA. The functional asymmetry of the substantia nigra striatum may contribute to the clinical unilateral symptoms of PD. However, a recent PET study on PD and MSA showed inconsistent findings, and the authors speculated that the previous conclusion may be confounded with selective bias (39). In our study, there were no differences in the left and right volumes of the putamen, caudate nucleus, and pallidum. The functional and structural asymmetry in PD and MSA may be related to the stage of disease progression, and further studies are warranted.

### Correlation between structural changes and clinical symptoms

UPDRS Part III and Part V (also known as modified H&Y classification) have been widely used in the clinical evaluation of PD. SPECT and PET studies on PD showed that the dopamine function in the substantia nigra striatum system was negatively correlated with UPDRS motor function and H&Y scores. Subsequent SPM analysis showed that the decrease of dopamine transporters was most remarkable in the posterior putamen (40). Recent morphological studies showed that UPDRS III has better reliability for predicting brain atrophy in early PD patients. In the present study, we demonstrated that the striatal nucleus volumes were not correlated with the UPDRS scores or the clinical course durations in PD patients. Therefore, we speculated that the decrease of dopamine uptake in the substantia nigra striatum system eventually leads to the occurrence of PD, whereas there is no change in striatal volume in the early stage of PD.

Tir et al. performed a VBM study on the motor circuit of MSA-P, which showed that H&Y scores were correlated with the volume of gray matter, white matter and putamen, and UPDRS III was correlated with the volume of putamen (4). However, another VBM study failed to find a correlation between UPDRS III and brain structure (27). Our study showed that the scores of UPDRS-III and modified H&Y scale were negatively correlated with the volume of the left putamen but not with the volume of other brain structures. These findings suggest that MSA is associated with the structural alterations in the nigra striatum system. Additionally, we speculate that the unilateral atrophy in the left putamen may be related to right-handedness. The positive correlation between the UPDRS-III score and the disease course indicates that the symptoms of MSA gradually become aggravated with disease progression. There was a significant negative correlation between the disease course and the volume of the bilateral putamen in patients with MSA-P, which revealed that the putamen was the earliest and most severely involved nucleus in MSA.

### VBM and ROI-based measurements

VBM can test and compare the whole brain and can be directly used in statistical analyses of original data without presetting the ROI. The density differences in brain tissue can be quantitatively identified. Furthermore, the method is characterized by automaticity, comprehensiveness, objectiveness, and repeatability. However, the application of VBM remains controversial because partial volume effects associated with tissue segmentation may be contaminated with other results, which may lead to errors. Voxel-based statistical analysis is premised on spatial normalization, and inaccurate matching of local areas and templates can lead to a system error. The T_1_ template of the MNI that is always used for VBM was established with young subjects and can lead to errors when used for older populations. Nevertheless, with continuous improvement, more recent versions of SPM (e.g., version 8) contain improved functional parameters to match the needs of VBM. VBM cannot be applied to the measurement of individual brain volume, which limits its clinical application. The classical ROI is the golden standard of structural measurements. However, the disadvantages of ROI, including time-consumption and operator dependence, have limited its range of applications in recent years. In this study, the results of two striatal volume measurements were consistent. Furthermore, we demonstrated that the bilateral putamen volume was smaller in the MSA-P patients compared with that in the PD patients using ROI; however, the VBM analysis failed to identify this difference. This finding indicates the superiority of the ROI measurement for microscopic structures. When VBM is used to compare striatal structures, we use AAL maps to optimize the volume of each nucleus. The ROI method yields results with high consistency.

## CONCLUSIONS

PD and MSA-P are associated with gray matter atrophy, which mainly involves the bilateral putamen, caudate nucleus, cerebellum, and temporal lobes. ROI and VBM can be used to identify these morphological alterations, and VBM is more sensitive and repeatable and less time-consuming, which may have potential diagnostic value.

## AUTHOR CONTRIBUTIONS

Dong C and Cui X were responsible for the study conception. Cui X and Li L were responsible for the data curation. Zhao L and Cui X were responsible for the formal analysis. Qian J and Song Q were responsible for the funding acquisition. Yu L, Xing H, Qian J and Chang H were responsible for the investigation. Zhou S was responsible for the methodology. Dong C was responsible for the project administration. Song Q and Dong C were responsible for the resources. Zhou S and Cui X were responsible for the software. Dong C was responsible for the supervision and validation. Cui X was responsible for the visualization and manuscript original writing. Cui X and Dong C were responsible for manuscript writing, review and editing.

## Figures and Tables

**Table 1:** Demographic characteristics of the patients with MSA-P and PD and the healthy controls.

	Patients with MSA-P (n=20)	Patients with PD (n=41)	Healthy controls (n=39)	Test statistics	*p* value
Male/Female	10/10	17/24	20/19	*X* ^2^=0.86	0.65^a^
Age (years)	66.25±8.82	65.22±8.82	62.82±6.23	*F*=1.54	0.22^b^
Education (years)	9.50±2.76	8.76±4.44	9.36±4.29	*F*=0.31	0.73^c^
Duration of disease (months)	41.4±22.6	33.71±30.39	-	*t*=1.00	0.32^d^
UPDRS (Part III)	40.08±15.12	32.15±7.78	-	*t*=2.21	0.04^e^
UPDRS (Part V)	2.98±0.91	2.23±0.54	-	*t*=3.38	0.01^f^

Values represent the mean ± SD.

a *p*>0.05, chi-square test, the three groups were gender matched.

b *p*>0.05.

c *p*>0.05, analysis of variance (ANOVA), age and education matched.

d *p*>0.05, t-test, the disease durations of MSA-P and PD were similar.

ef *p*<0.05, t-test, the patients with MSA-P had significantly increased scale scores compared with the patients with PD. UPDRS=Unified Parkinson’s Disease Rating Scale.

**Table 2:** Correlation analysis between clinical parameters and gray matter atrophy.

Diagnosis	Variables	Duration of disease	Gray matter atrophy						
			Putamen (left)	Putamen (right)	Caudate nucleus (left)	Caudate nucleus (right)	Globus pallidus (left)	Globus pallidus (right)	Whole brain gray matter
MSA-P									
	Duration of disease (*r* value*)	1.000	-0.577#	-0.722#	-0.057	-0.144	-0.345	-0.269	-0.325
	UPDRS (Part III) (*r* value)	0.597#	-0.592#	-0.399	0.076	0.168	0.190	0.145	0.050
	UPDRS (Part V) (*r* value)	0.328	-0.532#	-0.206	0.109	0.250	0.343	0.289	0.091
PD									
	Duration of disease (*r* value)	1.000	-0.118	-0.020	-0.170	-0.109	-0.181	-0.120	0.091
	UPDRS (Part III) (*r* value)	0.192	0.027	0.044	-0.182	-0.219	0.076	0.232	0.186
	UPDRS (Part V) (*r* value)	0.158	0.065	0.009	-0.098	-0.147	0.006	0.047	0.125

ICV and age set as controlled variables.

* *r* value (correlation coefficient)

# *p*<0.05.
